# Supplementary material for: Comparative study on corrosion characteristics of conductive concrete in red soil environment
Source: PLoS One. 2024 Dec 5;19(12):e0313256. doi: 10.1371/journal.pone.0313256 (PMC11620573; doi:10.1371/journal.pone.0313256)
Supplement: Attachment — Submitted filename: response to review.docx [file pone.0313256.s002.docx]

When submitting your revision, we need you to address these additional requirements.

1. Please ensure that your manuscript meets PLOS ONE's style requirements, including those for file naming. The PLOS ONE style templates can be found at 
https://journals.plos.org/plosone/s/file?id=wjVg/PLOSOne_formatting_sample_main_body.pdf and 
https://journals.plos.org/plosone/s/file?id=ba62/PLOSOne_formatting_sample_title_authors_affiliations.pdf

**Response: we have checked and modified the manuscript as the style requirements.**

2. Please note that funding information should not appear in any section or other areas of your manuscript. We will only publish funding information present in the Funding Statement section of the online submission form. Please remove any funding-related text from the manuscript.

**Response: the funding information have been deleted in manuscript.**

3. Thank you for stating the following financial disclosure: 
"This research was funded by the Key projects of Natural Science Foundation of Jiangxi Province under grant 2021ACB204004."

Please state what role the funders took in the study.  If the funders had no role, please state: "The funders had no role in study design, data collection and analysis, decision to publish, or preparation of the manuscript." 
If this statement is not correct you must amend it as needed. 
Please include this amended Role of Funder statement in your cover letter; we will change the online submission form on your behalf.

**Response: we have included this amended Role of Funder statement in your cover letter.**

4. In this instance it seems there may be acceptable restrictions in place that prevent the public sharing of your minimal data. However, in line with our goal of ensuring long-term data availability to all interested researchers, PLOS’ Data Policy states that authors cannot be the sole named individuals responsible for ensuring data access (http://journals.plos.org/plosone/s/data-availability#loc-acceptable-data-sharing-methods).

Data requests to a non-author institutional point of contact, such as a data access or ethics committee, helps guarantee long term stability and availability of data. Providing interested researchers with a durable point of contact ensures data will be accessible even if an author changes email addresses, institutions, or becomes unavailable to answer requests.

Before we proceed with your manuscript, please also provide non-author contact information (phone/email/hyperlink) for a data access committee, ethics committee, or other institutional body to which data requests may be sent. If no institutional body is available to respond to requests for your minimal data, please consider if there any institutional representatives who did not collaborate in the study, and are not listed as authors on the manuscript, who would be able to hold the data and respond to external requests for data access? If so, please provide their contact information (i.e., email address). Please also provide details on how you will ensure persistent or long-term data storage and availability.

**Response: we have upload our data in** **datadryad, it can be visit by https://doi.org/10.5061/dryad.crjdfn3dh**

**Reviewer #1: --- The following list of comments will help to further improve the manuscript:**
- The most striking section of a study is the abstract section. Therefore, important results from the study should be highlighted.

**Response: we have modified the abstract.**

- The novelty of the study should be mentioned a little more in the introduction section.

**Response: we have added a paragraph in introduction to address our novelty, which is highlighted.**
- The flowchart should be added to the study. Thus, the intelligibility of the study will increase.

**Response: that’s a good idea, and we have draw a diagram to show the diagram of our experiment, shown as Fig 5.**
- The “Grounding Metal Corrosion Evaluation Index and Test Method” and “Testing and Analysis of Corrosion Characteristics of Conductive Concrete Grounding Materials” sections should be rewritten more comprehensively.

**Response: we have added and revised in section 4.**
- The following sections should be rewritten carefully.
“4.2 Grounding Metals Corrosion Characteristics Testing in Different Environments The corrosion characteristics of commonly used grounding metals, Q235 carbon steel and galvanized steel, in red soil, ordinary concrete, and conductive concrete were investigated using the CS310H electrochemical workstation. Open-circuit potential, potentiodynamic polarization curves, and electrochemical impedance spectroscopy were conducted and analyzed for these metals in various environments. Different types and compositions of conductive concrete were also tested to evaluate their impact on the corrosion behavior of grounding metals. Fig 8 and Fig 9 depict the open-circuit potentials of Q235 carbon steel (comprising C, Mn, Si, S, P, and Fe) and galvanized steel (composed of common carbon steel with a zinc and aluminum coating). The polarization curves for Q235 carbon steel and galvanized steel can be seen in Fig 10 and Fig 11, while electrochemical impedance spectroscopy results are presented in Fig 12 and Fig 13. In these Figs, "SSF" refers to Stainless Steel Fiber.
4.3 Comparison of Metal Corrosion in Different Soil Environments From Fig 8 and Fig 9, it can be seen that the open-circuit potential of metal electrodes in red soil is lower than that in concrete, and the potential of specimens with conductive phase is higher than that of the ordinary concrete. The open circuit potential of metal samples was significantly increased whether they were wrapped in graphite based or stainless steel fiberbased conductive concrete. The results show that the corrosion sensitivity of metals in conductive concrete is greatly reduced. From the polarization curves in Fig 10 and Fig 11, it is evident that in red soil, the corrosion potential is the lowest and the corrosion current is the highest. In ordinary concrete, the corrosion potential increases and the corrosion current decreases compared to red soil. In conductive concrete, there is a noticeable increase in corrosion potential and a more significant decrease in corrosion current. Additionally, the passivation zone in red soil is notably shorter than in ordinary concrete and conductive concrete. This suggests that the abundance of acidic ions in red soil accelerates surface oxidation of the metal, making it challenging to form a stable passivation film. Therefore, wrapping the metal electrode with conductive concrete can potentially delay metal corrosion and enhance its durability. From the electrochemical impedance spectroscopy shown in Fig 12 and Fig 13, it is apparent that compared to concrete, the capacitance arc radius in red soil is very small, indicating poor corrosion resistance of grounding metals in a red soil environment. Furthermore, the capacitance arc radius in conductive concrete differs from that in ordinary concrete, depending on the specific conductive materials added and their concentration. In summary, among the four different soil environments tested, red soil exhibits the highest corrosion propensity, followed by ordinary concrete, while conductive concrete demonstrates good corrosion resistance. This difference arises due to the higher water and ion concentrations in red soil and the lower moisture and free ion concentrations in concrete, thereby weakening concrete's corrosion capability. Conductive concrete, enriched with conductive materials, serves to shunt current during experiments, thereby reducing electrolytic corrosion during electrification processes. 4.4 Comparison of Metal Corrosion in Different Conductive Concrete Based on the previous analysis, conductive concrete demonstrates effective corrosion protection. This material incorporates various conductive phase materials, with graphite representing non-metallic conductive phases and stainless steel fiber representing metallic conductive phases. Let's compare the effects of these different conductive phase materials on corrosion protection. In Figs 9 and 10, the open circuit potential of graphite conductive concrete is higher compared to stainless steel fiber conductive concrete. Additionally, the self-corrosion potential of graphite conductive concrete in Figs 10 and 11 is also higher than that of stainless steel fiber conductive concrete, indicating that graphite conductive concrete exhibits a smaller corrosion tendency and better corrosion resistance than stainless steel fiber. Moreover, in Figs 12 and 13, the radius of the capacitance arc for graphite conductive concrete is larger than that for stainless steel conductive concrete. This indicates that graphite conductive concrete offers greater impedance to corrosive charges and is less prone to corrosion. All three indicators consistently suggest that graphite conductive concrete provides superior corrosion resistance compared to stainless steel fiber conductive concrete. 4.5 Comparison of Metal Corrosion in Different Conductive Material Content In addition to the type of conductive phase materials, the content of these materials also significantly impacts the corrosion resistance of conductive concrete. Figs 8 to 13 illustrate that, for a given conductive phase material, increasing its content results in higher open circuit potential, polarization potential, and capacitance arc radius of the metal. These observations indicate that the corrosion resistance of conductive concrete improves as the content of conductive phase materials increases From a corrosion resistance perspective, higher concentrations of conductive phase materials positively affect the performance of conductive concrete. However, in practical applications, it is crucial to balance the content of conductive phase materials with considerations of mechanical strength and conductivity. Achieving an optimal ratio is essential to ensure both effective corrosion protection and satisfactory overall performance of conductive concrete. 4.6 Corrosion Comparison of Different Grounding Metals Comparing Fig 8 and Fig 9 under the same red soil environment, it is evident that the open circuit potential of galvanized steel has significantly increased compared to Q235 carbon steel. Upon stabilization, the potential becomes more positive, with the lowest value rising from - 0.083V to -0.0475V. This shift indicates lower corrosion sensitivity and stronger corrosion resistance for galvanized steel. In Figs 10 and 11, the self-corrosion current of galvanized steel is slightly positive compared to Q235 carbon steel, with a decrease of approximately two orders of magnitude in corrosion current. The passivation zone slope is also steeper and more pronounced for galvanized steel, suggesting that the zinc coating provides better corrosion resistance compared to Q235 carbon steel. Figs 12 and 13 show that the electrochemical impedance spectra of galvanized steel in red soil solution are generally similar to those of Q235 carbon steel. However, upon adding conductive phase materials, the capacitance arc radius of galvanized steel is slightly larger than that of Q235 carbon steel. These observations collectively indicate that galvanized steel exhibits better corrosion resistance than Q235 carbon steel across various environmental conditions.”
**Response: we have rewrited these sections as the content highlighted.**
- Lines in graphs should be thickened. (too bad)
**Response: we have thickened the lines from Fig 9 to Fig 14.**
- The “Conclusion” section should be reviewed. (It should be enriched with the results obtained.)

**Response: we have rewrited the conclusion.**

**Reviewer #2: Overall, this is a well-written manuscript. The introduction is relevant and theory-based, supported by experiments. Sufficient information about the previous study findings is presented for readers. The methods are generally appropriate Overall, the results are clear because of the good graphical representation.**

**Response: Thank you for your positive feedback and encouraging comments. Your recognition of our work is greatly appreciated.**
